# Differential activity of the antioxidant defence system and alterations in the accumulation of osmolyte and reactive oxygen species under drought stress and recovery in rice (*Oryza sativa* L.) tillering

**DOI:** 10.1038/s41598-019-44958-x

**Published:** 2019-06-12

**Authors:** Xinpeng Wang, Hualong Liu, Fengli Yu, Bowen Hu, Yan Jia, Hanjing Sha, Hongwei Zhao

**Affiliations:** Key Laboratory of Germplasm Enhancement, Physiology and Ecology of Food Crops in Cold Region, Ministry of Education, Northeast Agricultural University, Harbin, 150030 P. R. China

**Keywords:** Plant physiology, Drought

## Abstract

The objective of this study was to investigate the effects of drought stress on the activity of antioxidant enzymes and osmotic adjustment substance content in the tillering period of drought-sensitive and drought-tolerant rice cultivars. The results showed that the superoxide dismutase (SOD), peroxidase (POD), catalase activity (CAT), hydrogen peroxide content, soluble protein content and soluble sugar content increased with the accumulation of time and intensity of drought stress. Compared with the drought-sensitive cultivar, drought-resistant cultivar had a smaller photosynthetic affected area, longer CAT enzyme activity duration, and lower H_2_O_2_ accumulation. Unlike POD and CAT enzymes, which maintain the ability to scavenge hydrogen peroxide under long drought conditions, ascorbate peroxidase (APX) enzymes seem to be a rapid response mechanism to scavenge hydrogen peroxide under drought stress. Under a −10 kPa water potential, using soluble sugars on the osmotic adjustment ability of the drought-resistant cultivars was more efficient; under −40 kPa water potential, drought-resistant cultivars can maintain relative high levels of ascorbate (ASA) content in the short term. After the restoration of irrigation, the indices gradually returned to control levels. The ASA content showed faster accumulation ability in drought-resistant cultivars and faster recovery. The soluble protein content recovered more slowly in drought-sensitive cultivars under the −40 kPa treatment. Drought-resistant cultivars showed stronger resistance to drought in the −10 kPa treatment and obtained similar yield to the control, while the drought-sensitive cultivars were more obviously affected by the drought stress.

## Introduction

Drought has become the main reason for crop yield reductions in the world. As a major food crop, rice is also the most water intensive crop^1^. In the major rice production areas, the shortage of water resources has become the main problem in rice production. Therefore, it is very important to study water-saving cultivation in rice production. Drought reaction comprises changing their molecular, biochemical and physiological mechanisms and their morphology^2,3^. In the water sensitive stage, drought would weaken photosynthesis, growth and development would be inhibited, and the yield would be seriously affected^4^. Under the condition of water stress, the plant is directly or indirectly subjected to oxidative stress to cause cell membrane lipid peroxidation, a series of physiological and biochemical changes that can cause serious metabolic disorders and ultimately affect the yield and quality^5–7^. The antioxidant system played a key functional response to drought. Asada found the CO_2_ assimilation limit prior to the electron transfer passivation reaction. Therefore, the overproduction of the plant leads to the excessive reduction of photosynthetic electron chains and the generation of reactive oxygen species under drought stress^8^. These reactive oxygen species (ROS) such as superoxide anion (${{\rm{O}}}_{2}^{\cdot -}$), hydrogen peroxide (H_2_O_2_), and hydroxyl radical (HO⋅), they are produced in different organelles and cause oxidative damage to cell components, lipid and protein peroxidation, DNA fragmentation, enzyme inhibition, and activation of programmed cell death pathways, may eventually lead to cell death^9,10^. Mehdy M C considered that drought stress on plant damage was a result of intracellular free radical production and the elimination of imbalance^11^. It performed differently at different times under drought for rice. The activities of SOD, POD, CAT and soluble sugar in anthers of drought-tolerant rice cultivars increased, and the malondialdehyde (MDA) content decreased^12^. Drought during the filling period would increase the POD and CAT activity rapidly, while the SOD activity decreased slightly, the ASA and GSH contents decreased, and the H_2_O_2_ and MDA contents remained low. If the drought continued for some time, all indicators would decline markedly^13^. There are many studies about drought at the seedling stage of rice. It is generally believed that POD and CAT activity of leaves increased due to drought^14,15^. In plants, ascorbic acid is the most important reducing substrate for elimination of H_2_O_2_^16^. In the ascorbic acid-glutathione cycle, APX utilized two of ascorbic acid molecules to decompose H_2_O_2_ into water and this process is accompanied by the formation of monodehydroascorbate^17^. The enhancement of natural antioxidant components (enzymatic and non-enzyme) may be a strategy to reduce or prevent oxidative damage and improve plant drought resistance^18^.

However, the research on the antioxidant system of different drought-tolerant rice varieties under drought stress and re-watering in the tillering period is not clear. Therefore, we selected two rice cultivars with different comprehensive drought resistance index of rice screened in early studies for this experiment^19^. In the early study of drought stress, the difference of photosynthesis and nitrogen metabolism related traits between the two varieties also confirmed the difference of drought resistance between the two varieties^20,21^. In this study, the drought stress and re-watering effects on the antioxidant system of two drought-tolerant rice varieties at the tillering stage were studied in order to clarify the changes in the antioxidant system of drought-resistant and drought-tolerant varieties under different drought stress in the tillering period and reveal the physiological mechanism of drought tolerance in rice for further study.

## Results

### The effect of drought treatment on enzyme activity

**Figure 1:**
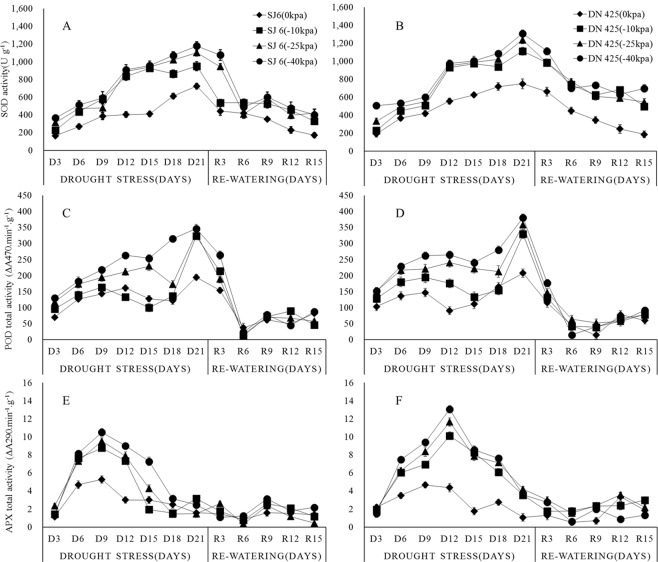
Effects of drought stress and re-watering on SOD (**A**,**B**), POD (**C**,**D**) and APX (**E**,**D**) activity in leaves at tillering stage of two rice cultivars in all treatments.SJ6, Songjing 6 (drought-sensitive); DN425, Dongnong 425 (drought-tolerant). The values represent the means ± SD (n = 3). The experiment was conducted at Northeast Agricultural University farm, Harbin, Heilongjiang Province, northeast China.

The activity of SOD increased with the prolongation of drought time, then reached a peak at 21 days after drought (Fig. 1A,B). Compared with the control, the peak of SOD activity of SJ6 treatments increased by 31.1%, 52.2%, 62.5%, respectively, while the SOD activity of DN25 treatments increased by 48.7%, 65.5%, 74.8%.

POD activity increased at the beginning of drought and then seemed to enter a platform stage (Fig. 1C,D). Except −40 kPa treatment of SJ6, the enzyme activity increased slowly after 6 days of drought, and even decreased from 9th to 15th day. The POD activity increased faster than before after 18 days and reached a peak at 21 days.

The activity of APX was increased rapidly after drought and reached the peak at 9th and 12th day in two cultivars, respectively (Fig. 1E,F). The activity was decreased rapidly after reached the peak. The activity of DN425 was higher than SJ6 in drought stress. Compared with the control, the peak of APX activity of SJ6 treatments increased by 66.3%, 80.8%, 99.3%, respectively, while the APX activity of DN425 treatments increased by131.8%, 166.7%, 199.2%.

**Figure 2:**
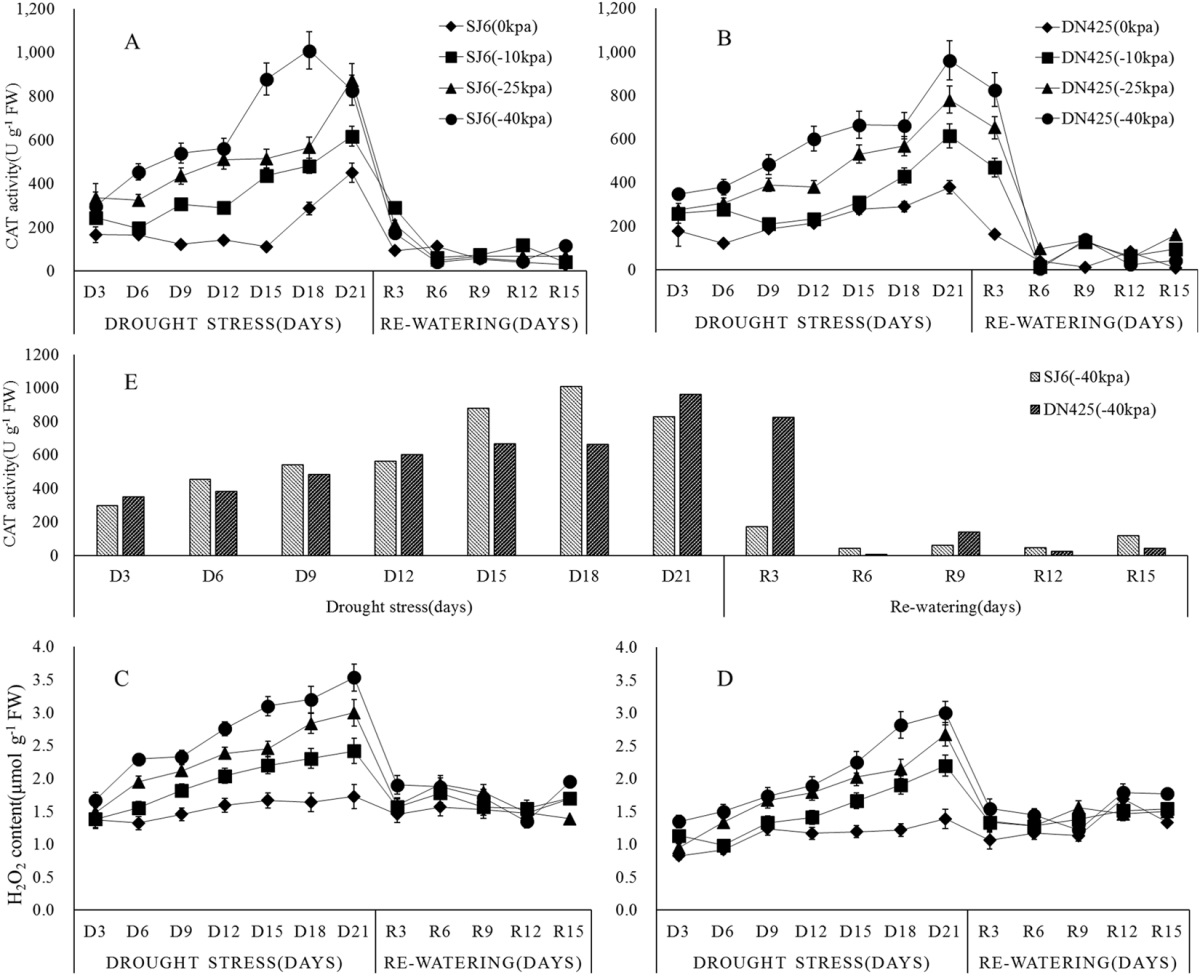
Effects of drought stress and re-watering on CAT activity (**A**,**B**) and H_2_O_2_ (**C**,**D**) content in leaves at tillering stage of two rice cultivars in all treatments. The CAT activity of SJ6 and DN425 in −40 kPa treatment (**E**). SJ6, Songjing 6 (drought-sensitive); DN425, Dongnong 425 (drought-tolerant). The values represent the means ± SD (n = 3). The experiment was conducted at Northeast Agricultural University farm, Harbin, Heilongjiang Province, northeast China.

CAT is an important enzyme used to eliminate H_2_O_2_. In this experiment, with the prolongation of drought time, the CAT activity in functional leaves of different treatments increased (Fig. 2A,B). Except for the −40 kPa treatment of SJ6, the CAT activity of the other treatments increased smoothly in 3–18 days after drought stress, then reached a peak at 21 days after drought. −40 kPa treatment of SJ6 showed a much higher CAT activity than the other treatments in 15–18 days after drought stress, the activity was 1.71 and 1.79 times more than the −25 kPa treatment of SJ6 at the same time. The CAT activity reached a peak at 18 days after drought stress in −40 kPa treatment of SJ6, three days earlier than the other treatments (Fig. 2,E). Compared with the control, the peak of SJ6 treatments increased by 36.8%, 94.5%, 124.3%, respectively, while the CAT activity of DN425 increased by 62.5%, 106.7%, 154.6%.

After 3 weeks of drought, the CAT activity of SJ6 decreased rapidly to the level of the third day of drought 3 days after the restoration of irrigation; the CAT activity of DN425 was only slightly decreased at the third day after the restoration of irrigation and was slightly higher than the level of day 18 of drought stress.

### The effect of drought treatment on H_2_O_2_ accumulation

Hydrogen peroxide is an important reactive oxygen. As shown in Fig. 2(C,D), the content of H_2_O_2_ gradually increased with the prolongation of drought stress, and the more severe the drought, the higher the content. The contents of H_2_O_2_ in the treatments reached a peak after 3 weeks of drought. Compared with the control, the H_2_O_2_ content of SJ6 treatments increased by 40.0%, 73.8%, 104.7%, respectively, while the content of DN425 treatments increased by 58.2%, 92.6% and 115.8%, respectively. In the −40 kPa treatments, the peak H_2_O_2_ content of DN425 was significantly lower than that of SJ6 (F = 7.816, F > F_0.05_).

The contents of H_2_O_2_ decreased rapidly in all treatments to early drought levels after the restoration of irrigation. The content of H_2_O_2_ in each treatment has a slight recovery at the end, probably due to the increase in ambient temperature.

### The effect of drought treatment on ASA accumulation

**Figure 3:**
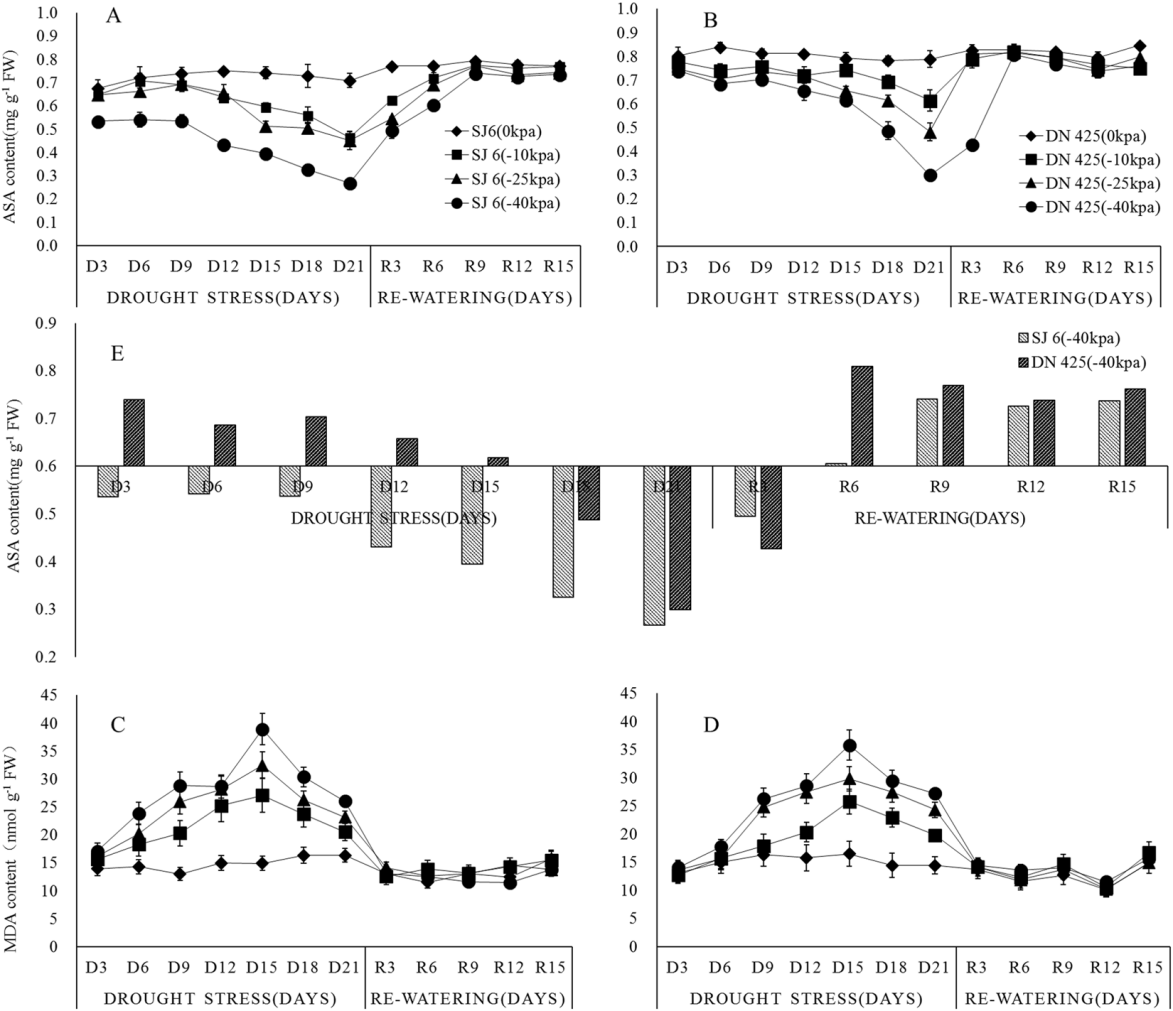
Effects of drought stress and re-watering on ASA (**A**,**B**) and MDA (**C**,**D**) content in leaves at tillering stage of two rice cultivars in all treatments. The ASA content of SJ6 and DN425 in −40 kPa treatment (**E**). SJ6, Songjing 6 (drought-sensitive); DN425, Dongnong 425 (drought-tolerant). The values represent the means ± SD (n = 3). The experiment was conducted at Northeast Agricultural University farm, Harbin, Heilongjiang Province, northeast China.

ASA is an important antioxidant, playing a vital role in the elimination of H_2_O_2_. The results showed that the ASA content in functional leaves decreased first and then increased, and all ASA contents decreased to the lowest point after 3 weeks of drought (Fig. 3A,B). Compared with the control, the ASA content of SJ6 treatments decreased to 65.5%, 63.7%, and 37.7%, and DN425 treatments decreased to 78.0%, 61.2% and 38.0%. It is noteworthy that the ASA contents of SJ6 and DN425 did not show a significant decrease in the first time, except for the A3 treatment (Fig. 3,E). The ASA content in functional leaves of SJ6 and DN425 declined during the drought after 9 days and 12 days, respectively. The ASA content of the A3 treatment was significantly lower than that of the other treatments since the 3rd day of drought and decreased further after 9 days of drought until it dropped to the lowest point after 3 weeks of drought.

After the restoration of irrigation, the ASA content increased rapidly in all treatments, and the content of ASA in functional leaves of SJ6 increased to the control level on the 9th day after the restoration of irrigation. The ASA content in DN425 returned to the control level on the third day after the resumption of irrigation, while the B3 treatment returned to the control level on the 6th day.

### The effect of drought treatment on MDA accumulation

Excessive accumulation of MDA can lead to cell membrane lipid peroxidation. The results showed that (Fig. 3C,D), compared with the control, the MDA content increased as the time and intensity of drought increased. The MDA content in all treatments reached a peak on the 15th day of drought and then decreased gradually. Compared with the control, the peak of MDA content of SJ6 treatments increased by 81.6%, 117.5% and 160.7%, respectively, while the MDA content of DN425 treatments increased by 73.2%, 100.4% and 140.3% respectively.

After the restoration of irrigation, the MDA content in all treatments continued to decline and reached a minimum on the 3rd day after the restoration of irrigation. The MDA content did not change significantly, which was close to the control level.

### The effect of drought treatment on soluble protein accumulation

**Figure 4:**
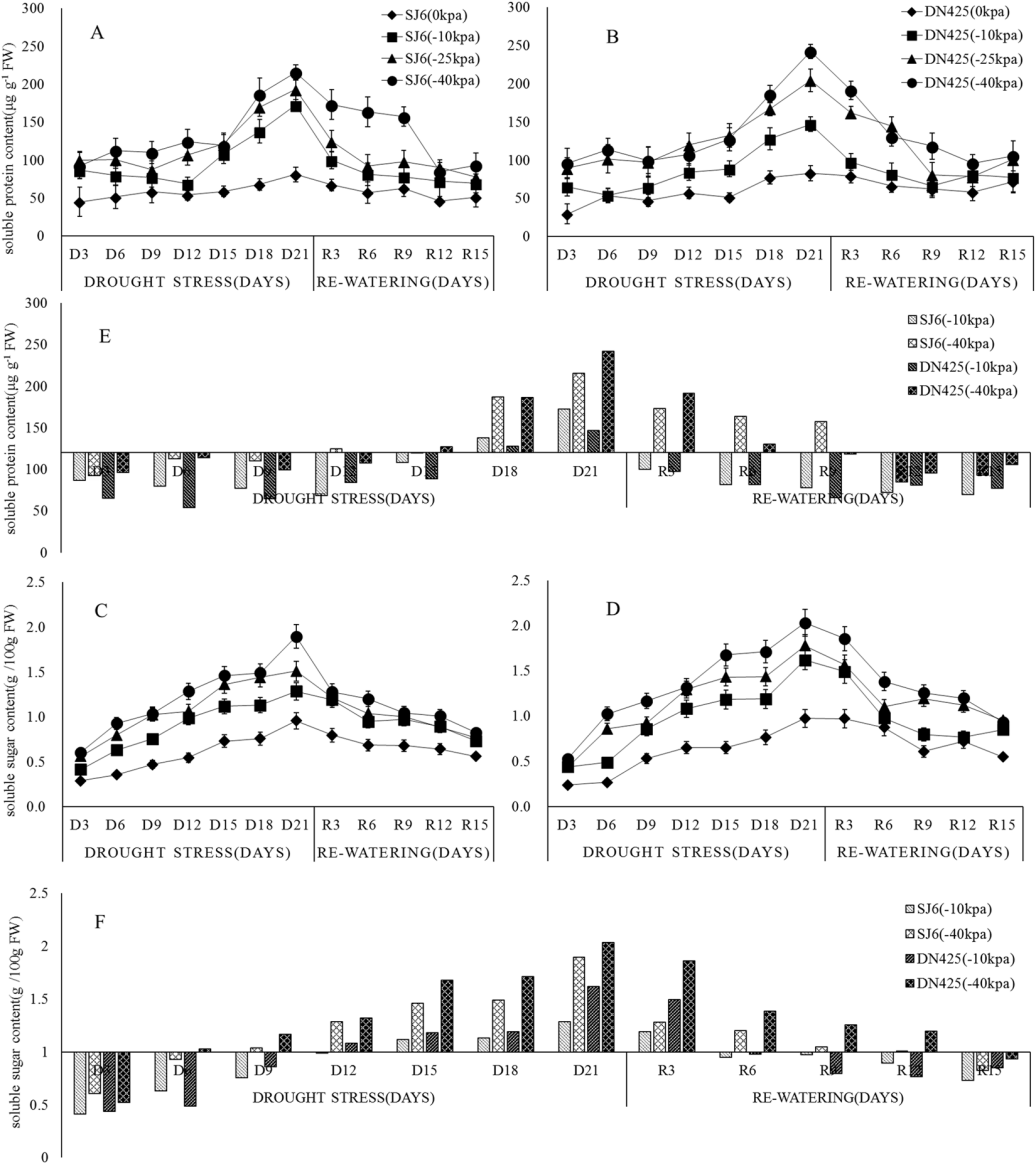
Effects of drought stress and re-watering on Soluble protein (**A**,**B**) and Soluble sugar (**C**,**D**) content in leaves at tillering stage of two rice cultivars in all treatments. The Soluble protein (**E**) and Soluble sugar (**F**) content in −10 kPa and −40 kPa treatment of SJ6 and DN425. SJ6, Songjing 6 (drought-sensitive); DN425, Dongnong 425 (drought-tolerant). The values represent the means ± SD (n = 3). The experiment was conducted at Northeast Agricultural University farm, Harbin, Heilongjiang Province, northeast China.

Soluble protein is an important component of cell osmotic regulation. As shown in Fig. 4(A,B), the content of soluble protein increased with the prolongation of drought time, and the higher the drought intensity, the higher the soluble protein content. The level of soluble protein content of all treatments reached a peak at 3 weeks of drought. Moreover, the soluble protein content of DN425 was significantly higher than that of SJ6 under the −40 kPa treatment (F = 7.772, F > F_0.05_), while under the −10 kPa treatment, it was significantly lower than that of SJ6 (F = 8.452, F > F_0.05_) (Fig. 4,E). Compared with the control, the peak of soluble protein content of SJ6 treatments increased by 112.4%, 138.3%, 165.8%, respectively, while the content of DN425 treatments increased by 76.8%, 146.1% and 191.5%, respectively.

After the restoration of irrigation, the soluble protein content of all treatments decreased to the level of the initial stage of drought. Under the −40 kPa treatment of SJ6, the content of soluble protein was not significantly decreased at 3–9 days after recovery, then, the soluble protein content decreased to the level of initial drought. Compared with SJ6, the content of soluble protein of DN425 in the −40 kPa treatment decreased rapidly to the control level after the restoration of irrigation.

### The effect of drought treatment on soluble sugars accumulation

Soluble sugars and soluble proteins are important osmotic regulators. As shown in Fig. 4(C,D), the soluble sugar content of each treatment showed a single peak curve, which first increased and then decreased, and the higher the drought intensity, the higher the content. The contents reached the highest value after 3 weeks of drought, and the peak of DN425 was significantly higher than that in SJ6 in −10 kPa treatment (F = 11.373, F > F_0.05_) (Fig. 4,F). Compared with the control, the peak of soluble sugars content of SJ6 treatments increased by 34.2%, 57.7%, 98.0%, while the content of DN425 treatments increased by 66.3%, 82.8% and 108.7%.

After the restoration of irrigation, the soluble sugar contents in all treatments began to decline. During the three days after the restoration of irrigation, the soluble sugar content of SJ6 decreased rapidly, while that of DN425 decreased slowly in −40 kPa treatment.

### The effect of drought treatment on ${{\bf{O}}}_{{\bf{2}}}^{\cdot -}$ production

**Figure 5:**
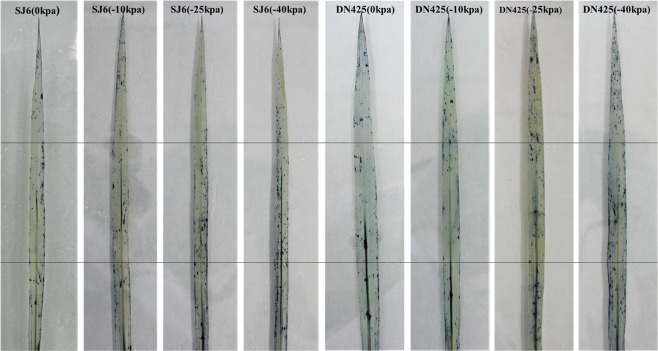
Histochemical localization of ${{\rm{O}}}_{2}^{\cdot -}$ in top leaf at tillering stage of two rice cultivars in all treatments after three weeks drought stress. *Dark blue deposits* show insoluble formazan produced by the reaction of NBT with ${{\rm{O}}}_{2}^{\cdot -}$.

The top leaf of two cultivars was stained with NBT after 3 weeks of drought, and the results showed that the superoxide anion accumulation increased with the increase in drought intensity (Fig. 5). Superoxide anions gradually accumulated in the middle of leaves of SJ6, the accumulation of one-third regions in the top of the leaf is less. In the −40 kPa treatment, accumulation in the base can also be clearly seen. The superoxide anion accumulation was mainly in the tip of the leaf of DN425, and the accumulation in the middle part of the leaf was relatively small. The superoxide anion also substantial accumulated in the middle and the base of leaf in the −40 kPa treatment. It is noteworthy that compared to the control, DN425 did not have significant differences in −10 kPa treatment. Moreover, DN425 had less accumulation than SJ6 in the middle and the base of leaf in −10 kPa treatment.

### Main effect test and correlation coefficients for different indexes, yield of two rice cultivars under drought treatment

Various characters responded differently to different varieties and treatments. The main effect analysis was used to identify whether drought stress or varietal variation was responsible for these characters. As shown in Table 1, the drought stress had a significant effect on the indices. The differences of the varieties had no significant effect on the CAT activity and soluble protein content, and the effect on the other indices was significant or extremely significant. From the analysis of variance of varieties and treatments, the contents of soluble protein, soluble sugar and H_2_O_2_ were significantly affected by drought stress, while the CAT activity and MDA and ASA contents depended on the varieties and treatments.

**Table 1:** Main effect test for CAT activity and content of soluble protein (SP), MDA, H_2_O_2_, ASA and soluble sugar (SS) in leaves of rice between treatments and varieties (F values).

	CAT	SP	MDA	H_2_O_2_	ASA	SS
Treatment (*T*)	16.533**	331.244**	65.102**	71.702**	60.597**	393.292**
Varieties (*V*)	0.145	0.166	15.595**	4.349*	52.078**	47.078**
*T* × *V*	0.058	4.225**	0.886	4.202**	1.501	5.502**

*Significant at P < 0.05.

**Significant at P < 0.01.

The correlation analysis of characters was used to check whether there are positive or negative correlations among these characters. Table 2 shows that the soluble sugar content and CAT activity during drought stress and after the restoration of irrigation showed highly significant negative correlations with the ASA content and were significantly positively correlated with other indices.

**Table 2:** Correlation coefficients among different indexes in leaves of rice under drought stress and recovery at tillering stage.

	CAT	SP	MDA	H_2_O_2_	ASA	SS
CAT	1					
SP	0.711**	1				
MDA	0.495**	0.159	1			
H_2_O_2_	0.671**	0.793**	0.128	1		
ASA	−0.777**	−0.761**	−0.330**	−0.677**	1	
SS	0.678**	0.853**	0.279**	0.800**	−0.624**	1

*Significant at P < 0.05.

**Significant at P < 0.01.

As shown in Table 3, there was no difference in the yields of SJ6 and DN425 under normal irrigation, and the yield of DN425 was significantly higher than that of SJ6 under the same water potential. Different drought stress significantly low the yield of SJ6, while the yield of B1 was lowed not significantly, compared with the control.

**Table 3:** Yield of SJ6 and DN425 under drought treatment.

	CK/g m^−2^	−10 kPa/g m^−2^	−25 kPa/g m^−2^	−40 kPa/g m^−2^
SJ6	729.18 a	628.04 c	576.99 e	492.12 f
DN425	743.36 a	715.50 a	680.31 b	603.72 d

Alphabets indicates differences between drought treatments values (P ≤ 0.05).

## Discussion

Mehdy M C found that intracellular free radical generation and drought stress clear imbalances were the main factors causing plant damage^11^. Different scholars believed that antioxidant enzyme activity increased in rice under drought stress (Wang H Z *et al*., 2007). In this experiment, the activity of CAT in drought-sensitive cultivar SJ6 began to decrease before the return to irrigation under long-term drought at −40 kPa. CAT is the primary enzyme responsible for the removal of H_2_O_2_. CAT activity, which declined earlier, also led to a certain degree of accumulation in the H_2_O_2_ content, and the peak H_2_O_2_ content under the −40 kPa treatment of SJ6 that was significantly higher than that of DN425 also proved this point. we hypothesized that APX was a rapid response mechanism of rice to reduce H_2_O_2_ under drought stress at the tillering stage. For rice that suffered drought at the tillering stage, the APX mechanism started first and quickly reduced the H_2_O_2_, while at the same time, the slower clearance mechanisms of POD and CAT started. When the activity of POD and CAT reached a certain level, the APX activity decreased and gradually transitioned the work of reduced H_2_O_2_ to the two enzymes. We also observed the activity of APX did not change significantly when the CAT activity of SJ6 decreased in −40 kPa treatment, while the POD activity was significantly higher than that of other treatments, which can be seen from accumulation of H_2_O_2_, their common substrates. The fact that the peak of H_2_O_2_ accumulation of drought-sensitive cultivar SJ6 was significantly higher than drought-tolerant cultivar DN425 but was still in a cell within an acceptable range. Therefore, we hypothesized that at this time, the activity of POD increased to compensate for the lack of H_2_O_2_ scavenging capacity because of the decrease in CAT activity. After the drought stress was removed, the CAT activity of DN425 did not decrease as rapidly as in SJ6. As a protective enzyme in the antioxidant system, it remained for a relatively long time with higher activity, which is significant for the further reduction in the accumulation of H_2_O_2_ during drought stress to maintain a lower level.

ASA is considered a powerful antioxidant because of its ability to donate electrons in a number of enzymatic and nonenzymatic reactions. It plays an important role in several physiological processes in plants, including growth, differentiation, and metabolism (Sharma *et al*. 2012). As the substrate for APX, in the ascorbate-glutathione cycle, two molecules of ascorbic acid are utilized by APX to eliminate H_2_O_2_ to water with the concomitant generation of monodehydroascorbate^17^. In this study, we observed that the ASA content of SJ6 and DN425 decreased 9 d and 12 d after drought stress, respectively. Coincidentally, the peak of APX activity appeared 9 d and 12 d after drought, respectively, and then decreased rapidly, which showed that APX activity was regulated by the ASA content. Early in the drought, the ASA content did not decrease significantly. Therefore, we speculated that the ASA used to eliminate H_2_O_2_ was in the dynamic equilibrium of consumption and synthesis at this time; until the CAT enzyme activity increased to a certain level, then lowed the content of ASA to complete the work transfer of H_2_O_2_ elimination. The difference between −10 kPa and −25 kPa treatments of SJ6 was not significant, but the ASA content in the −40 kPa treatment was significantly lower than that of other treatments. The possible reason was that the higher H_2_O_2_ content caused the higher ASA content required for reduction. However, there was no decrease in the ASA content at the beginning of drought stress in DN425. Therefore, it was suggested that maintaining a high ASA content under severe drought was one of the reasons for its drought resistance. The maintenance of a high antioxidant capacity to scavenge ROS has been linked to increased tolerance of plants to a wide range of environmental stresses^9,22^. Our research confirms this view. After relieving the drought, the ASA content began to accumulate, and that of DN425 accumulated more rapidly, which was vital for the recovery of metabolic function after the drought.

There are many ways to produce H_2_O_2_ in plant cells, a considerable part of which is produced by disproportionation of ${{\rm{O}}}_{2}^{\cdot -}$ by SOD, and the accumulation of ${{\rm{O}}}_{2}^{\cdot -}$ is the main reason for cell lipid peroxidation. The top leaf of rice was stained with NBT after 3 weeks of drought, and ${{\rm{O}}}_{2}^{\cdot -}$ accumulation and ${{\rm{O}}}_{2}^{\cdot -}$ accumulation parts were slightly different between DN425 and SJ6. As the degree of drought strengthened, the content of ${{\rm{O}}}_{2}^{\cdot -}$ was mainly accumulated in the middle and base of the leaves of SJ6, and the accumulation of ${{\rm{O}}}_{2}^{\cdot -}$ in the tip was relatively small; ${{\rm{O}}}_{2}^{\cdot -}$ mainly accumulated in one-third regions in the top of DN425, resulting in a greater affected photosynthetic area in SJ6 than that in DN425. Under −40 kPa, the ${{\rm{O}}}_{2}^{\cdot -}$ accumulation of two cultivars was higher than that of other treatments. Compared with the control, it was noteworthy that the ${{\rm{O}}}_{2}^{\cdot -}$ accumulation was not significant under −10 kPa treatment of DN425, which may also be one of the reasons for its drought resistance. Photosynthesis is the most important activity for plant growth. The smaller affected photosynthetically area meant a stronger assimilation ability and more photosynthetic products, which was very important for plants under stress.

During drought stress, the osmotic pressure of the cells played a key role in maintaining the water potential of the plant^23^. Soluble protein, soluble sugar and proline are important for cellular osmotic adjustments. During drought stress, the relative water content of a plant decreases by 60–80%, with an increase in the osmotic potential of the plant cells^24^. This increases the level of osmolytes and ensures that the plant maintains its water content during drought, enabling the plant to sustain its growth and yield^25^. In this experiment, the contents of soluble protein and soluble sugar, which accumulated under drought stress and decreased after drought relief, were similar, and the content of the soluble protein and soluble sugar showed a significant positive correlation, which indicated that they have synergistic effects in the cell osmotic adjustment under drought. In −40 kPa treatment of SJ6, the content of soluble protein was not significantly decreased at 3–9 days (R3~R9) after recovery, and the possible reason was a long period of severe drought that cannot be quickly restored and still needs a certain level of osmotic adjustment substances to stabilize the osmotic pressure. An interesting phenomenon is that the contents of the soluble protein and soluble sugar were different between two cultivars in −10 kPa treatments. The content of soluble protein was significantly lower in DN425 than SJ6 under the −10 kPa treatment with 21 days drought stress, while the soluble sugar content was significantly higher than SJ6. In the previous discussion, we knew that the photosynthetically affected area of DN425 was smaller than SJ6 in −10 kPa treatment. Therefore, we boldly speculated that the result of difference in two varieties osmotic adjustment was DN425 used the photosynthetic products of soluble sugar to maintain the osmotic pressure balance, while the soluble protein was used as a supplement in the cell loss of water in −10 kPa treatment. This explains why the soluble sugar content of DN425 in −10 kPa treatment was significantly higher than that of SJ6. SJ6 had a larger affected photosynthetic area and less photosynthetic products, which meant that the soluble sugar was also less than that of DN425 and was forced to use a large amount of soluble protein to make up for this deficiency, resulting a significantly higher soluble protein content than that of DN425. After drought relief, the soluble protein content of SJ6 did not decrease rapidly at −40 kPa, while its soluble sugar content decreased rapidly, which may also be the reason.

Yield is the most important indicator of a crop. In this experiment, DN425 maintained a significantly higher yield than SJ6 under the same treatment, and the loss in yield of SJ6 with the increasing intensity of drought was very serious, while the yield loss of DN425 was less than that of SJ6. Compared with the control, the yield did not change significantly under the −10 kPa treatment, which is a common result of different physiological controls under drought stress.

Results show that different drought stresses on the antioxidant system in rice were caused by different degrees of influence, and the drought-resistant variety DN425 showed CAT activity that was maintained for a long time. In the −10 kPa treatment, the affected photosynthetically area is smaller, and the use of soluble sugar on the ability of cells to perform osmotic adjustment is stronger. In the −40 kPa treatment, DN425 can maintain higher levels of ascorbic acid in the short term. Compared with the drought-sensitive cultivar SJ6, the drought-resistant cultivar DN425 showed lower H_2_O_2_ accumulation under drought. After the restoration of irrigation, DN425 showed a faster recovery ability for the ASA content and showed a higher soluble sugar content in the short time after restoration of irrigation. The recovery of the soluble protein content was slower in the drought-sensitive cultivar SJ6 under the −40 kPa treatment. The yield of DN425 in the −10 kPa treatment was similar to the control, while that of SJ6 was more obviously affected by drought stress.

## Materials and Methods

### Experimental site and design

The present research was conducted at the A’Cheng experimental site of the Northeast Agricultural University in Harbin City from June to October in 2016. The experiment was conducted with two rice cultivars with different drought tolerances^19–21^. Songjing 6 (SJ6) has low drought resistance, 135 days (d) growth duration, and 2500 °C active accumulated temperature, approximately. Dongnong 425 (DN425) has medium drought resistance, 140 days (d) growth duration and 2550 °C accumulated temperature, approximately. The average values for the selected soil characteristics of composite topsoil samples (0–20 cm) from the main experimental plots were as follows: organic matter was 20.34 ± 0.34 g kg^−1^; total N was 1.52 ± 0.09 g kg^−1^; total P was 0.49 ± 0.06 g kg^−1^; slowly available K was 654.5 ± 4.34 mg kg^−1^; available N, 1 mol L^−1^ NaOH-alkali-hydrolysed N was 129.8 ± 4.34 mg kg^−1^; available P, 0.5 mol L^−1^ NaHCO_3_-Olsen P was 16.3 ± 0.5 mg kg^−1^; available K, 1 mol L^−1^ NH_4_OAc-exchangeable K was 89.4 ± 1.1 mg kg^−1^; and the pH was 6.45 ± 0.07. Seedlings were sowed on 18 April and transplanted on 30 May with a hill spacing of 30 cm × 10 cm and three plants per hill. The fertilization standard was composed of nitrogen (150 kg per ha as urea), phosphorus (100 kg per ha as diammonium phosphate) and potassium (75 kg per ha as potassium sulphate). Urea was also used at mid-tillering (100 kg per ha) as a top dressing.

The experiment was designed with three gradients of soil water potential (−10 kPa, −25 kPa, −40 kPa) and a randomized blocks experimental design, with a well-watered treatment (0 kPa) as the control. The drought stress treatment restored irrigation after drought three weeks. The initial date (June15) of the drought stress is defined as the date on which all treatment reaches the design water potential (Fig. S1). The irrigation was controlled before the drought stress to gradually low the soil water potential, and a small amount of water was added to maintain potential at the design if there was a water shortage in the near stress period. All treatments were under a rain-proof shelter during the drought treatment period. The soil potential was monitored daily at 6 am, 12 am and 6 pm with a soil tensiometer (Institute of Soil Science, Chinese Academy of Sciences), three times the average value of the soil potential on behalf of the day. Normal irrigation was restored immediately after the drought stress.

The physiological parameters, including the activities of SOD, POD, APX and CAT, the content of soluble protein, MDA, H_2_O_2_, ASA, and soluble sugar were measured once every 3 days after the start of stress.

### Measurement items and methods

#### Extraction and determination of enzyme activities

A 0.5 g leaf sample was ground with a mortar and pestle and homogenized in liquid nitrogen in 5 mL of a 50-mM sodium phosphate buffer (pH 7.0) containing 1 mM EDTA-Na_2_ and 4% (w/v) polyvinylpyrrolidine-40 (PVP-40). The homogenate was centrifuged at 10,000 × g for 20 min at 4 °C.SOD activity was measured based on the method of Beauchamp and Fridovich^26^. POD activity was measured based on the method of Upadhyaya^27^. CAT activity was measured according to the method of Aebi^28^. APX activity was detected according to the method of Mittler and Zilinskas^29^.

#### The content of soluble protein, MDA, H_2_O_2_, ASA and soluble sugar

The soluble protein concentration was analysed according to Bradford^30^ using Coomassie Brilliant Blue G-250 (Sigma) as dye and albumin (Bovine V; Sigma) as the standard. The MDA concentration was analysed according to Heath and packer^31^. A reaction mixture containing 2 mL supernatant, 1 mL TCA 20% (w/v), and 0.5% thiobarbituric acid was incubated at 100 °C in a water bath for 30 min, and then cooled immediately before centrifugation. Absorbance of the supernatants was determined at 450, 532, and 600 nm. Calculation of MDA content was based on the following formula: C (μm L^−1^) = 6.45 × (OD_532_ − OD_600_)−0.56 × OD_450_.

The concentration of H_2_O_2_ was determined according to Brennan and Frenkel^32^. A 0.5 g leaf sample was ground in 5 mL of refrigerated acetone. The homogenate was centrifuged at 10,000 × g for 10 min at 4 °C. 1 mL of the supernatant was mixed with 0.1 mL 5% Ti(SO_4_)_2_ and 0.2 mL ammonia. After the precipitate was formed, the reaction mixture was centrifuged at 10,000 × g for 10 min at 4 °C. The resulting pellet was dissolved in 2 M H_2_SO_4_, and the absorbance was measured at 415 nm. The H_2_O_2_ level was calculated according to a standard curve of H_2_O_2_.

A 1.0 g leaf sample was ground in liquid nitrogen with a mortar and pestle and homogenized in 5 mL 5% TCA. The homogenate was centrifuged at 4,000 × g for 10 min at 4 °C, and the supernatant was assayed for reduced form ASA concentration. 1 mL of the supernatant was mixed with 1 mL 5% TCA and 1 mL ethanol and shaken, and 0.5 mL 0.4% H_3_PO_4_- ethyl alcohol solution, 1 mL 0.5% BP (2,9-Dimethyl-4,7-diphenyl-1,10- phenanthroline)- ethyl alcohol solution and 0.5 mL 0.03% FeCl_3_- ethyl alcohol solution were added, with a total volume of 5 mL. The solution was allowed to react for 90 min at 30 °C, and the absorbance was measured at 534 nm. The reduced form ASA level was calculated according to a standard curve of ASA^33^.

Soluble sugar was determined based on Anthrone Colorimetry. 5 mL of distilled water were added to 0.2 g fresh leaves, extracted twice in boiling water for 30 min each time and the constant volume of the extract was 25 mL. 0.5 mL of the extract was mixed with 0.5 mL anthrone – ethyl acetate and 5 mL 98% sulfuric acid, with heat preservation in boiling water for 1 min immediately, and then it was cooled to room temperature; The extract was replaced with distilled water in the blank. The absorbance was measured at 630 nm. The soluble sugar level was calculated according to a standard curve of soluble sugar.

#### *In situ* histochemical monitoring of ${{O}}_{{2}}^{{\cdot }-}$ production in drought-stressed rice organs

*In situ* histochemical monitoring of ${{\rm{O}}}_{2}^{\cdot -}$ production in the leaves was performed with NBT^34^. The selected rice leaves were first soaked for 4−16 h in the PBS containing NBT (0.05%) and NaN_3_ (10 mM) at 37 °C. Then, these leaves were transferred into ethanol solution and incubated for 30 min at 70 °C until blue spots appeared on them. The chlorophyll in the leaves was removed using ethanol washings 4−5 times.

### Statistical analysis

Data analyses were performed using the SPSS 18.0 (Chicago, IL) software package. Analysis of variance (ANOVA) was adopted to analyze all data and differences among treatment. Results are reported as the mean ± standard deviation (SD) values of the three independent experiments, measuring at least three different replicates(plants) in each experiment. SD was calculated directly from crude data. Levels of significance in figures are given by ns, *, ** for not significant, significant at P < 0.05 and P < 0.01, respectively.

## Supplementary information

Figure S1

## References

[1] Shuxing, L. I. *et al*. The Responding of Rice after Water Stress in Young Panicle Formation Stage. *ACTA Agriculturae Boreali-Sinica* (2014).

[2] Yousfi S, Marquez AJ, Betti M, Luis Araus J, Dolores Serret M (2016). Gene expression and physiological responses to salinity and water stress of contrasting durum wheat genotypes. Journal Of Integrative Plant Biology.

[3] Chaves MM, Maroco JP, Pereira JS (2003). Understanding plant responses to drought - from genes to the whole plant. Functional Plant Biology.

[4] Yao Lin ZH, Jianxia L, Hui H (2014). Huang Huang. Effects of water stress at tillering stage on rice growth and development and yield of rice under different cultivation modes. Crop Research.

[5] Wei A, Wang Z, Chen B, Zhai Z, Zhang Y (2004). Effect of soil drought on electron transport rate and photophosphorylation level of different green organs in wheat. Acta Agronomica Sinica.

[6] Lanceras JC, Pantuwan G, Jongdee B, Toojinda T (2004). Quantitative trait loci associated with drought tolerance at reproductive stage in rice. Plant physiology.

[7] Chaves MM, Oliveira MM (2004). Mechanisms underlying plant resilience to water deficits: prospects for water-saving agriculture. Journal of experimental botany.

[8] Asada K (1999). The water-water cycle in chloroplasts: Scavenging of Active Oxygens and Dissipation of Excess Photons. Annual Review of Plant Physiology & Plant Molecular Biology.

[9] Sharma, P., Jha, A. B., Dubey, R. S. & Pessarakli, M. Reactive Oxygen Species, Oxidative Damage, and Antioxidative Defense Mechanism in Plants under Stressful Conditions. *Journal of Botany,2012,(2012-4-24)* **2012** (2012).

[10] Sharma, P., Jha, A. B. & Dubey, R. S. In *Handbook of plant and crop stress* 109–158 (CRC press, 2016).

[11] Mehdy MC (1994). Active Oxygen Species in Plant Defense against Pathogens. Plant physiology.

[12] Fu G-f (2011). Changes of Oxidative Stress and Soluble Sugar in Anthers Involve in Rice Pollen Abortion Under Drought. Stress. Agricultural Sciences in China.

[13] Liu SH, Chen GX, Yin JJ, Lu CG (2011). Response of the Flag Leaves of a Super-Hybrid Rice Variety to Drought Stress during Grain Filling Period. Journal of Agronomy and Crop Science.

[14] Lum MS, Hanafi MM, Rafii YM, Akmar ASN (2014). Effect of drought stress on growth, proline and antioxidant enzyme activities of upland rice. Journal Of Animal And Plant Sciences.

[15] Li X, Zhang L, Li Y (2011). Preconditioning Alters Antioxidative Enzyme Responses in Rice Seedlings to Water Stress. Procedia Environmental Sciences.

[16] del Rio LA, Sandalio LM, Corpas FJ, Palma JM, Barroso JB (2006). Reactive oxygen species and reactive nitrogen species in peroxisomes. Production, scavenging, and role in cell signaling. Plant physiology.

[17] Shao HB, Chu LY, Shao MA, Jaleel CA, Mi HM (2008). Higher plant antioxidants and redox signaling under environmental stresses. Comptes rendus biologies.

[18] Hasanuzzaman, M., Nahar, K., Gill, S. S. & Fujita, M. *Drought Stress Responses in Plants, Oxidative Stress, and Antioxidant Defense*. (Wiley‐VCH Verlag GmbH & Co. KGaA, 2013).

[19] Wang, Q. *et al*. Selection of Drought-resistant Rice Variety(Lines) in Heilongjiang Province. *Heilongjiang Agricultural Sciences* (2009).

[20] Meifang, Y. U. Effect of Drought Stress at Tillering Stage on Photosynthetic Characteristics and Yield Formation of Cold-region Rice. *Journal of Nuclear Agricultural Sciences* (2017).

[21] Zhao, H. *et al*. Effect of drought stress at booting stage on grain nitrogen formation and yield of rice in cold-region. *Journal of Northeast Agricultural University* (2017).

[22] Mishra P, Bhoomika K, Dubey RS (2013). Differential responses of antioxidative defense system to prolonged salinity stress in salt-tolerant and salt-sensitive Indica rice (Oryza sativa L.) seedlings. Protoplasma.

[23] Osakabe Y, Osakabe K, Shinozaki K, Tran LSP (2014). Response of plants to water stress. Frontiers in plant science.

[24] Singh R, Pandey N, Naskar J, Shirke PA (2015). Physiological performance and differential expression profiling of genes associated with drought tolerance in contrasting varieties of two Gossypium species. Protoplasma.

[25] Blum A (2005). Drought resistance, water-use efficiency, and yield potential—are they compatible, dissonant, or mutually exclusive?. Australian Journal of Agricultural Research.

[26] Beauchamp C, Fridovich I (1971). Superoxide dismutase: improved assays and an assay applicable to acrylamide gels. Analytical Biochemistry.

[27] Upadhyaya A, Sankhla D, Davis TD, Sankhla N, Smith BN (1985). Effect of Paclobutrazol on the Activities of some Enzymes of Activated Oxygen Metabolism and Lipid Peroxidation in Senescing Soybean Leaves. Journal of plant physiology.

[28] Aebi H, Aebi H (1984). Catalase *in vitro*. Methods in Enzymology.

[29] Mittler R, Zilinskas BA (1993). Detection of ascorbate peroxidase activity in native gels by inhibition of the ascorbate-dependent reduction of nitroblue tetrazolium. Analytical Biochemistry.

[30] Bradford MMA (1976). A Rapid and Sensitive Method for Quantitation of Microgram Quantities of Protein Utilizing the Principle of Protein-Dye Binding. Analytical Biochemistry.

[31] Heath RL, Packer L (1968). Photoperoxidation in isolated chloroplasts.i. kinetics and stoichiometry of fatty acid peroxidation. Archives of Biochemistry and Biophysics.

[32] Brennan T, Frenkel C (1977). Involvement of Hydrogen Peroxide in the Regulation of Senescence in Pear. Plant Physiology.

[33] Arakawa N, Tsutsumi K, Sanceda NG, Kurata T, Inagaki C (1981). A rapid and sensitive method for the determination of ascorbic acid using 4,7-diphenyl-1,10-phenanthroline. Agricultural & Biological Chemistry.

[34] Rossetti S, Bonatti PM (2001). *In situ* histochemical monitoring of ozone- and TMV-induced reactive oxygen species in tobacco leaves. Plant Physiology & Biochemistry.

