# Supplementary material for: Differential activity of the antioxidant defence system and alterations in the accumulation of osmolyte and reactive oxygen species under drought stress and recovery in rice (Oryza sativa L.) tillering
Source: Sci Rep. 2019 Jun 12;9:8543. doi: 10.1038/s41598-019-44958-x (PMC6561971; doi:10.1038/s41598-019-44958-x)
Supplement: Supplementary file 1 — Figure S1 [file 41598_2019_44958_MOESM1_ESM.docx]

Differential activity of the antioxidant defence system and alterations in the accumulation of osmolyte and reactive oxygen species under drought stress and recovery in rice (*Oryza sativa* L.) tillering

Xinpeng Wang^&^, Hualong Liu^&^, Fengli Yu, Bowen Hu, Yan Jia, Hanjing Sha, Hongwei Zhao^*^

Rice Research Institute, College of Agriculture, Northeast Agriculture University, 600 Changjiang Road, Harbin 150030, Heilongjiang, China

& These authors contributed equally to this work.

* Corresponding author. Tel.: +0086 0451 55190292.

E-mail: hongweizhao_cool@126.com (H. Zhao).


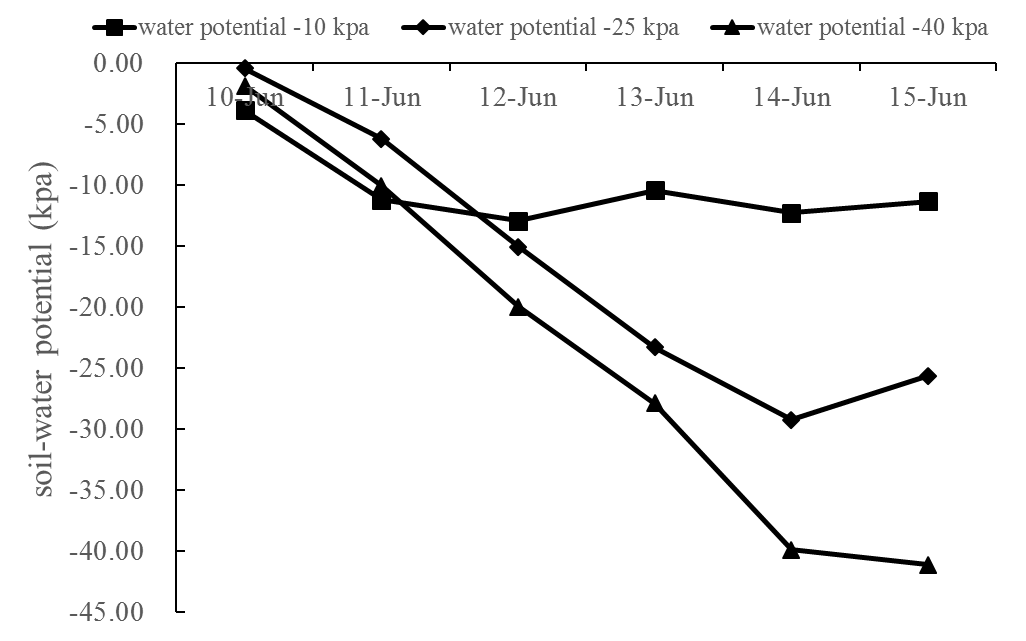


Fig. S1. Soil water potential was monitored and controlled before the drought stress
